# CRAFTING COLLABORATIONS THAT BENEFIT STUDENTS, CLIENTS, AND COMMUNITY

**DOI:** 10.1093/geroni/igae098.2696

**Published:** 2024-12-31

**Authors:** Stacey Gandy, C J Golding, Gillian Bryant

**Affiliations:** Arizona State University, Phoenix, Arizona, United States; Arizona State University, Tempe, Arizona, United States; Arizona State University, Phoenix, Arizona, United States

## Abstract

When a university partners with a low-income residency for older adults, the results are exponential. A student-run, interdisciplinary drop-in center housed within a Section 8 apartment building for older adults and adults with disabilities arose from such a partnership. This program provides interdisciplinary service-learning opportunities for students while serving the needs of the residents. Social work students work alongside other disciplines, such as nursing, recreational therapy, nutrition, program planning, poetry, and music, to support residents’ emotional, social, psychological, and physical well-being. In the 2022-2023 academic year, the program served 257 of the building’s 300 residents and provided learning opportunities for 300 students. A 2013 needs assessment of the building’s residents found considerable loneliness, social isolation, and disconnectedness. Healthy Places by Design (2021) recommends intergenerational community centers that prioritize socialization among older adults and younger people, which is a major focus of the drop-in center. Students foster interaction with and between residents to encourage community connectedness. Research demonstrates that social interaction between older adults and college students can result in decreased loneliness for older adults and decreased attitudes of ageism in students (Ramamonjiarivelo et al., 2022). As our aging population grows, so does the importance of preparing social work students to work with older adults. We are excited to share the ways in which bringing older adults and student interns together is mutually beneficial and provides an innovative solution to eradicate loneliness, social isolation, and community disconnectedness in older adults and ageism in students.

